# The Library.—I

**Published:** 1912-08-03

**Authors:** 


### RELAXATIONS AND HOBBIES.

### The Library.—I.

Among the votaries of medicine there have always been found those who choose a hobby of an intellectual nature. It may be connected with their work—something which brings the reflection that professional pursuits are not being allowed *zu einer Erwerbsquelle herabsinken*. On the other hand, it may be purely recreational, a pleasant interlude varying the routine of prescribing stomachic sedatives and operating on hæmorrhoids. The former joy is for those who are made of stern stuff. Nowadays single-handed research is an arduous business; in the words of Plato, the doer must be at the beck of the thing to be done. Conceive, for instance, the case of an unfortunate individual set the task of exploring every yard of a railway system from rail head to central terminus, each succeeding branch having to be traced out in all its widely diverging ramifications. Almost generic with this is the lot of the worker who, in order to get complete knowledge of a subject, sets out to traverse the mazes of modern medical literature. The beginning is easy enough: an ordinary text-book reference points to a few original papers which are either classical or likely to become so. But these, when consulted, refer to German archives, to French theses, Italian polyclinics, Hungarian, or even Russian, medical journals; and when hard dictionary work overcomes at length the polygot difficulty, each further reference leads on to others—"and so" (almost) "*ad infinitum*." Then, when thus pushed to its confines, the study of the subject is likely enough to call for pretty exact knowledge of some branch of physical science, of statistics, of demography, of political economy; or of all these. The demand is, of course, not likely to be fully satisfied in the case of most of us. The claims of the *Erwerbsquelle* aforesaid prevent this, let alone our not unlimited powers. Nevertheless, as scientific and artistic workers alike are aware, to pursue a worthy aspiration educates as nothing else can, forming at the same time an elevating, if strenuous, pleasure.

### The Pursuit of Culture.

Those who love nearly any sort of knowledge for its own sake are greatly in the minority in this country, even, one may say, in the learned professions themselves. Irrationally enough, the public school tradition hugely exalts what is known as "character," which is just the ordinary Anglo-Saxon's strongest point, at the expense of intellect, which is just where he comes off worst. Not long ago, a novelist extracted some effective comedy from this fact. He represented a young Russian, sent to pass his last term or two at an English public school, being asked by the master to read a chapter of some Old Testament prophet. In all innocence the lad amiably begs to be allowed to read instead from his own copy of Dostoieffsky, citing as his reason opinions held by advanced Biblical scholars. Now how many British medical students are in sympathy at all with this youth's predilections, or, indeed, have much comprehension of his point of view? Nevertheless, a good deal may be done to correct the present fashionable roughness and indifference to things of the mind, and instances can be culled from events recently occurring in our own profession. In not very distant days army medical officers had the name of affecting an aristocratic and ultra-military tone, and of disclaiming any keenness for technical work. Now, owing partly to the advance of tropical medicine, all that has been changed, and manifold good has resulted. In the profession generally, too, although the clinician still probably gets quite as much notice and admiration as is good for him, yet the research worker has risen greatly both in the estimation of his confrères and of the public. Let it be hoped that in our hospital schools the idol of the international football field and the crack examination passer may shed a few of their laurels on him who tries with some success to stand (to borrow Tolstoy's striking phrase in *What is Art?*) on the height of the knowledge of our day.

### The British Museum Reading-Room.

In any mention of libraries, there is only one possible to begin with, and that is the reading-room of the British Museum, access to which, as is well known, is guarded by special permission. Tourists and children perambulate the sculpture galleries, but they may not cross the hall to the passage leading to the reading-room. To obtain admission it is necessary to apply in writing to the Director, specifying profession or business, place of abode, and the particular purpose for which admission is sought. No person will be admitted for the purpose of preparing for examination or of writing prize-essays, unless on special reason being shown. The application must be made two days at least before admission is required, and has to be backed by a written recommendation from a householder. Armed with the ticket, which it is well to get initialled for the manuscripts room, one passes the guardians of the portal after parting with stick or umbrella, and enters the famous apartment.

It is a great circular chamber, roofed in by a windowed dome. Below the glass are inscribed more or less notable names in English literature, the selection being apt to excite remark because Macaulay's is blazoned with equal distinction to that of Shakespeare. Below the names again, all round the walls, are ranged 20,000 books. To such a circumference the readers' desks radiate from the centre of the floor, where are found the counters of the collecting and distributing staff, fenced in by low shelves of subject and of author indices. To obtain books one fills up requisition slips with the aid of these indices, and perhaps also of some special bibliographical compilation, from which latter may be made the astonishing and dismaying discovery that there do exist works not included even in this grand collection. One then puts the slips in a collecting box, and retires to the seat one has indicated on it. Here there will be about half an hour to wait, and as no man of taste can insult the genius of the place by reading a newspaper, the best thing to do is to stroll to the wall and take down the first volume one sees. Very soon then comes the shock of realising the immensity of learning garnered in this place. The subject unexpected, the author by way of being an authority (for books frequently referred to are kept here), his paragraph or two one reads rings impressive like a strange voice. Replacing the book, the reader finds his eye wander along the adjacent rows of serried backs. But . . . . of course . . . ! there must be tens of thousands of such paragraphs in each of the hundreds and hundreds of thousands of books here!

The student must, however, be back at his seat in good time to receive his books from the hand of the attendant. They are admirable reading desks, with extra shelves, a comfortable chair, and plenty of elbow room. One of one's neighbours will very likely be an old man. Evidently, in spite of Talleyrand's advice, a tolerable old age can be passed browsing on literature, making erudite entries (to receive equally erudite responses) in the suggestion book; an old age probably quite independent of the recreation derivable from cards. There recur to one, however, the words of the philosopher Reid, whose sad experience was that trying to acquire fresh information in old age is like pouring water into a vessel already full to the brim. On one's other hand may be a journalist exploring the files of some forgotten magazine of last century: further along the line, a couple of ladies defy with feminine impunity the rule of silence that obtains in all reading-rooms. But the books arrive, and seemingly a little magically, a little as though one were Aladdin. For, although humanist literature is much better represented than scientific (compare, say, the relative numbers of publications on heraldry and on heredity), yet the resources available here are vast, and to see actually before one in leather and paper some work the name of which—so remote as it seemed—one disinterred from a bulky index, is certainly a little thrilling. A couple of hours pass, and one bears one's volumes back. It is the excellent rule to return them personally at the central counter when done with, and to receive back the requisition slips in exchange.

(*To be concluded.*)

